# The joint effects of loneliness and interpersonal abuse on suicidal thoughts and behaviors among young adults in higher education in the United States

**DOI:** 10.1186/s40359-025-03184-z

**Published:** 2025-07-30

**Authors:** Hans Oh, André Hajek, Jinyu Du, Lee Smith, Edouard Leaune, Louis Jacob

**Affiliations:** 1 Suzanne Dworak Peck School of Social Work, University of Southern California, Los Angeles, CA USA; 2 University of California, Berkeley, CA USA; 3 Department of Health Economics and Health Services Research, Hamburg Center for Health Economics, University Medical Center Hamburg-Eppendorf, Hamburg, Germany; 4 Southern Methodist University, Dallas, TX USA; 5 Centre for Health, Performance, and Wellbeing, Anglia Ruskin University, Cambridge, UK; 6 Hospices Civils de Lyon, Lyon, France; 7 Research on Healthcare Performance RESHAPE, INSERM U1290, Université Claude Bernard Lyon 1, Lyon, France; 8 Université Paris Cité, Assistance Publique – Hôpitaux de Paris (AP-HP), Lariboisière-Fernand Widal Hospital, Department of Physical Medicine and Rehabilitation, Paris, 75010 France; 9 Université Paris Cité, INSERM, UMR U1153, Epidemiology of Ageing and Neurodegenerative Diseases, Paris, 75010 France; 10 Research and Development Unit, Parc Sanitari Sant Joan de Déu CIBERSAM, ISCIII, Dr. Antoni Pujadas, 42, Sant Boi de Llobregat, Barcelona, Spain

**Keywords:** Interpersonal abuse, Loneliness, Suicide, Suicidal ideation, Suicide attempt, Violence, United states

## Abstract

**Background:**

Loneliness and interpersonal abuse are two factors that contribute to suicide risk, but studies exploring their interactions on suicidal thoughts and behaviors are lacking. Thus, our aim was to examine potential synergies between loneliness and interpersonal abuse among young adults in higher education.

**Methods:**

We analyzed data from the Healthy Minds Study (2020–2021; *N* = 101,744) and used multivariable logistic regression models to show the interaction between loneliness (UCLA 3-item Loneliness Scale) and interpersonal abuse (verbal, physical, and/or sexual) on suicidal thoughts and behaviors (suicidal ideation, suicide plans, suicide attempts), adjusting for age, gender, race/ethnicity, depression, anxiety, and food insecurity.

**Results:**

Those who only reported being lonely had significantly greater odds of suicidal ideation (aOR: 3.01; 95% CI: 2.72–3.33), and those who only reported interpersonal abuse also had greater odds (aOR: 2.97; 95% CI: 2.52–3.50), when compared to those who reported neither. However, those who endorsed both loneliness and interpersonal abuse had the greatest odds (aOR: 5.65; 95% CI: 5.09–6.27), exceeding the sum of these individual effects. A similar and more pronounced pattern emerged for suicide plans and suicide attempts.

**Conclusion:**

A synergy exists between loneliness and interpersonal abuse in relation to suicidal thoughts and behaviors among young adults in higher education.

**Supplementary Information:**

The online version contains supplementary material available at 10.1186/s40359-025-03184-z.

## Introduction

Suicide is an enduring societal problem, costing the United States around $510 billion every year [42]. Among United States (US) young adults, suicide is a leading cause of death, and suicidal thoughts and behaviors (STB) seem to be increasing. From 2009 to 2015, the 12-month prevalence of suicidal ideation rose from an estimated 6.1 to 8.3% among US young adults [22]. Similarly, the 12-month prevalence of suicide plan rose from 2.0 to 2.7%, and the 12-month prevalence of suicide attempt rose from 1.1 to 1.6% [22]. According to the Center for Disease Control and Prevention (2017), over a thousand college students die by suicide annually across campuses, calling for more research on suicide risk factors in this population. Several frameworks have been developed to explain STB, and these frameworks point to a range of psychosocial and environmental risk factors, such as mental pain and interpersonal problems [20]. Among the many risk factors, this study focuses on loneliness and interpersonal abuse as risk factors related to STB, which are noted in the literature [30, 38].

The Interpersonal Theory of Suicide (ITS) states that suicide is a result of thwarted belongingness, perceived burdensomeness, and acquired capability for suicide [10, 27, 49]. Here we view loneliness as a reflection of thwarted belongingness and perceived burdensomeness. We also view interpersonal abuse as a potential mechanism by which people may acquire the capability for suicide, where the interpersonal abuse may decrease physical pain sensitivity and diminish fear of suicide-related actions and lower fear of death [5].

### Loneliness

Loneliness is defined as the subjective distressing experience that results from perceiving one is socially isolated or that one has inadequate meaningful social connections [8]. According to the Healthy Minds Monthly Poll, in 2024, 30% of adults aged 18–34 said that they were lonely several times a week or every day. Loneliness has been associated with numerous psychosocial problems (e.g., self-esteem, social competence), mental health problems (e.g., depression, anxiety), physical health problems (e.g., cardiovascular conditions, headaches), behavioral health problems (e.g., drug use, eating problems, sleep disturbances), and multimorbidity [24, 46]. Several studies have demonstrated robust associations between loneliness and STB, including one meta-analysis that showed loneliness is a major factor contributing to STB in prospective longitudinal studies in various populations [35]. Loneliness is related to STB across various contexts and populations [9].

### Interpersonal abuse

Interpersonal abuse has also been strongly related to STB. Experiences of abuse, including childhood maltreatment, intimate partner violence, and workplace harassment, have been consistently linked to increased risk of STB [1, 15, 23]. A meta-analysis found that childhood maltreatment was strongly associated with STB in adulthood [2], which some studies suggest may operate via impulsivity [6], low self-esteem [19], and other mental health pathways. For instance, interpersonal abuse is also related to psychiatric disorders, including depression, post-traumatic stress disorder, and substance use [28], which are strong drivers of suicide.

### Current study

When loneliness and exposure to interpersonal abuse come together, they may form a vicious cycle, where being socially isolated can make one more likely to be targeted for interpersonal abuse; at the same time, interpersonal abuse can be socially isolating and can cause feelings of loneliness [39, 44]. We also consider the vulnerability stress model [40], which suggests psychological factors (in this case, loneliness) can be moderated by negative life events (including traumatic experiences, stressors, and situational factors) to impact risk for STB. To our knowledge, there are few if any studies that have examined the extent to which loneliness and interpersonal abuse interact to increase odds of STB among large diverse samples of young adults in higher education. Considering this gap, we examine this topic.

## Methods

### Sample

We analyzed data from the 2020–2021 Healthy Minds Study (HMS), a non-probability web-based survey examining health and wellness among students enrolled in higher education in the United States (details about the HMS annual survey are described elsewhere, such as Lipson et al., [33]. The HMS survey is administered annually as a repeated cross-section of schools, with a different set of schools every year, including community colleges, four-year colleges, and professional schools. The HMS survey uses several validated measures to provide information about the prevalence of mental health outcomes, knowledge and attitudes about mental health, and service utilization. During 2020–2021, the survey was administered at 140 institutions of higher learning September 2020 and June 2021. Students were incentivized to participate by being entered into raffles to receive prizes upon completion of the survey. The total response rate was 14%, which is comparable to other response rates from online surveys using convenience samples and panels [12]. We restricted the sample by age (18–29) to isolate young adults and further excluded individuals who were missing data on any of the variables of interest. The total sample was *N* = 130,566 (the average age was 23); however, we used complete-case analysis, resulting in a final analytic sample of 101,744 (average age was 21). To account for non-response, sample probability weights were developed using administrative data from the full student populations at each participating college. Using multivariable logistic regression, response propensity was estimated using gender, race/ethnicity, academic level, and Grade Point Average. We then assigned response propensity weights to each student who completed the survey, such that students who were less likely to have completed the survey were assigned a larger weight in the analysis. The weighting scheme assigned each participating school equal aggregate weight in the national estimates, preventing larger institutions from disproportionately influencing overall national figures. Standard errors were clustered at the school level to account for the nested structure of the data and potential within-institution correlation.

### Ethics approval and consent to participate

The HMS data collection was approved by the Institutional Review Board Advarra (IRB number: Pro00028565), and the Institutional Review Boards at all participating campuses. All participants provided informed consent to participate. This secondary analysis was approved by the Institutional Review Board at the University of Southern California (UP-22-00068).

### Data Availability

A list of all participating schools as well as the HMS data are available upon request at: https://healthymindsnetwork.org/hms/.

### Measures

#### Suicidal thoughts and behaviors (past year)

STB was measured using items adapted from the World Health Organization suicide screen. Suicidal ideation was measured by the dichotomous item (yes/no): “In the past year, did you ever seriously think about attempting suicide?” Respondents who stated ‘yes’ were then asked two additional items: “In the past year, did you make a plan for attempting suicide? (yes/no)” and “In the past year, did you attempt suicide? (yes/no)”.

#### Loneliness

Loneliness was measured using the 3-item UCLA Loneliness Scale [26], where respondents were asked three questions: “How often do you feel that you lack companionship?”; “How often do you feel left out?”; “How often do you feel isolated from others?”. Respondents could answer hardly ever, some of the time, or often. These items were summed into a scale ranging from 3 to 9, with greater scores, suggesting more loneliness. This scale was dichotomized to reflect people who were significantly lonely (i.e., who had scores of 6 or higher) in accordance with prior studies [26].

#### Interpersonal abuse (past year)

Interpersonal abuse was measured using three dichotomous (yes/no) items administered in the HMS (see details about the HMS survey in Lipson et al., [33]: (1) Over the past 12 months, were you kicked, slapped, punched or otherwise physically mistreated by another person?; (2) Over the past 12 months, were you called names, yelled at, humiliated, judged, threatened, coerced, or controlled by another person?; and (3) In the past 12 months, has anyone had unwanted sexual contact with you? (Please count any experience of unwanted sexual contact [e.g., touching of your sexual body parts, oral sex, anal sex, sexual intercourse, and penetration of your vagina or anus with a finger or object] that you did not consent to and did not want to happen regardless of where it happened). Recent abuse was coded to reflect the presence of at least one type of abuse.

##### Covariates

Sociodemographic characteristics and mental health (covariates). We restricted the sample to focus on young adults (aged 18–29 years) and further controlled for age as a continuous variable. We also adjusted for gender (cis-gender man, cis-gender woman, transgender/nonbinary/other), and race/ethnicity (White, Black, Latine/Hispanic, Asian American/Pacific Islander, multiracial, and other). Food insecurity was assessed using two items, which asked: (1) Within the past 12 months I was worried whether our food would run out before we got money to buy more; (2) Within the past 12 months the food I bought just didn’t last and I didn’t have money to get more. Respondents could answer: never true, sometimes true, often true. Individuals were identified as food insecure with an affirmative answer (sometimes true or often true) to either question, following the two-item screen for families at risk of food insecurity [21]. Depression was measured using the Patient Health Questionnaire – 9 (PHQ-9;Kroenke & Spitzer [31],. The scale ranged from 0 to 27, which was dichotomized (scores 15 and higher) to reflect moderately severe to severe depression. *Anxiety* was measured using the General Anxiety Disorder – 7 (GAD-7; Spitzer et al., [45]. The scale ranged from 0 to 21 and was dichotomized (scores 11 and higher) to reflect moderately severe to severe anxiety.

#### Analysis

We calculated the prevalence of loneliness, interpersonal abuse, and STB, along with all covariates (total and stratified by STB). We tested for moderation using an additive scale and depict the synergy between loneliness and interpersonal abuse on STB (suicidal ideation, suicide plans, and suicide attempts) by creating the following categorical variable: (1) neither loneliness nor interpersonal abuse; (2) loneliness only; (3) interpersonal abuse only; and (4) both loneliness and interpersonal abuse. We adjusted for age, gender, race/ethnicity, food insecurity, depression, and anxiety. We calculated the interaction contrast ratios (ICRs), which allows use of odds ratios derived from logistic models to estimate the relative excess risk resulting from the synergy of combined exposures [50]. Confidence intervals and P-values for ICRs were generated using the nlcom command in Stata SE 15. Figures were generated using ChatGPT.

## Results

The sample characteristics of the HMS have been detailed in prior studies [41]. Over 60% of the sample was White, roughly 57% were women, with a mean age of 21. Table 1 provides the descriptive statistics for the analytic sample (Tables S1 and S2 in Supplemental Materials provide additional descriptive statistics). Over the past 12 months, about 14.2% of the sample reported suicidal ideation, 5.9% reported suicide plan, and 1.5% reported suicide attempt. Over half (56.6%) of the sample reported being lonely, and almost a third (32.9%) reported having at least one type of abuse (i.e., physical, emotional, and/or sexual abuse).

**Table 1:** Descriptive statistics for the healthy minds study 2020–2021 (*N* = 97762)

	Total *n* (%)	No suicide attempt (%)	Suicide attempt (%)	*P*-value
**Age** (18–29 years old; mean)	21.16	21.17	20.45	< 0.001
**Gender**				< 0.001
Man	38,444 (39.32%)	38,013 (39.46%)	431 (30.12%)	
Woman	56,063 (57.35%)	55,206 (57.31%)	857 (59.89%)	
Transgender/Nonbinary/Other	3255 (3.33%)	3112 (3.23%)	143 (9.99%)	
**Race/ethnicity**				< 0.001
1 (White)	59,416 (60.78%)	58,690 (60.93%)	726 (50.73%)	
2 (Asian Pacific Islander)	9161 (9.37%)	9019 (9.36%)	142 (9.92%)	
3 (Black)	10,623 (10.87%)	10,387 (10.78%)	236 (16.49%)	
4 (Hispanic)	7735 (7.91%)	7619 (7.91%)	116 (8.11%)	
5 (Two or more)	9429 (9.64%)	9238 (9.59%)	191 (13.35%)	
6 (Other)	1398 (1.43%)	1378 (1.43%)	20 (1.40%)	
**Food insecurity (past 12 months)**				< 0.001
Not food insecure	67,806 (69.36%)	67,218 (69.78%)	588 (41.09%)	
Food insecure	29,956 (30.64%)	29,113 (30.22%)	843 (58.91%)	
**Depression (past 2 weeks)**				< 0.001
No	75,304 (77.03%)	74,831 (77.68%)	473 (33.05%)	
Yes	22,458 (22.97%)	21,500 (22.32%)	958 (66.95%)	
**Anxiety (past 2 weeks)**				< 0.001
No	62,409 (63.84%)	62,013 (64.37%)	396 (27.69%)	
Yes	35,353 (36.16%)	34,319 (35.63%)	1034 (72.31%)	
**Loneliness (past 2 weeks)**				< 0.001
No	42,413 (43.38%)	42,220 (43.83%)	193 (13.50%)	
Yes	55,349 (56.62%)	54,112 (56.17%)	1237 (86.50%)	
**Any abuse**				< 0.001
No	65,604 (67.11%)	65,238 (67.72%)	366 (25.58%)	
Yes	32,158 (32.89%)	31,093 (32.28%)	1065 (74.42%)	
**Sexual abuse**				< 0.001
No	90,215 (92.28%)	89,230 (92.63%)	985 (68.83%)	
Yes	7547 (7.72%)	7101 (7.37%)	446 (31.17%)	
**Physical abuse**				< 0.001
No	91,209 (93.30%)	90,175 (93.61%)	1034 (72.26%)	
Yes	6553 (6.70%)	6156 (6.39%)	397 (27.74%)	
**Verbal abuse**				< 0.001
No	69,374 (70.96%)	68,915 (71.54%)	459 (32.08%)	
Yes	28,388 (29.04%)	27,416 (28.46%)	972 (67.92%)	

*P*-values reflect chi-square tests for binary and dichotomous variables, and t-tests for continuous variables

**Fig. 1:**
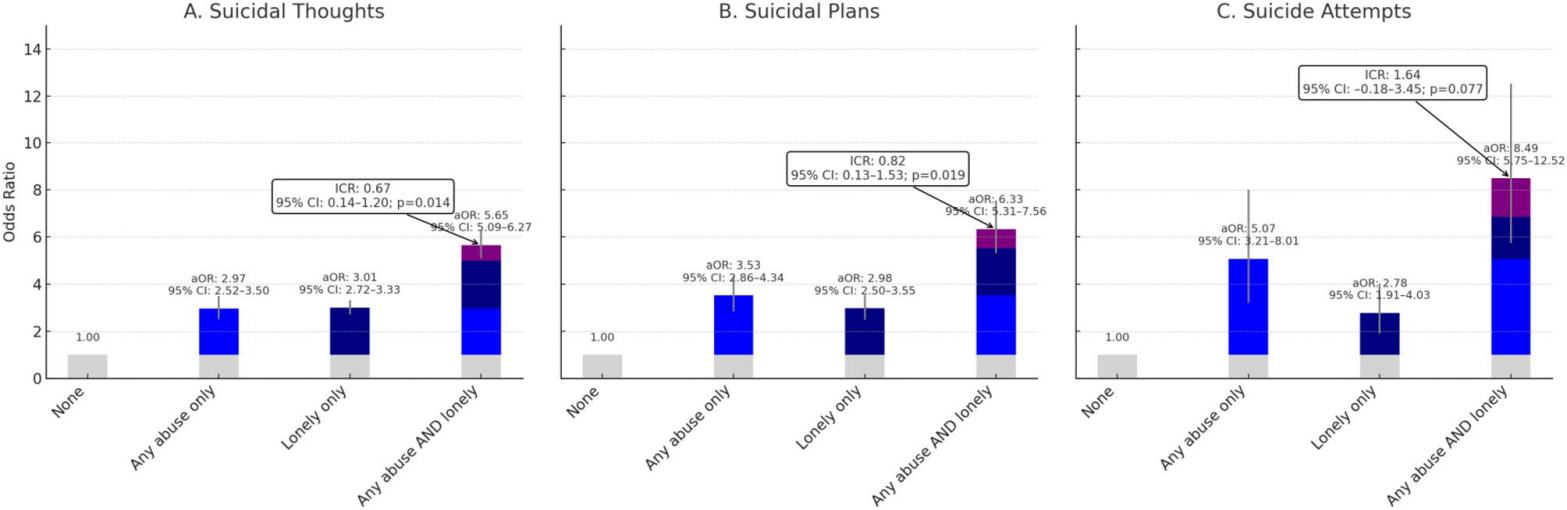
Multivariable logistic regression showing separate and combined odds of loneliness and interpersonal abuse on suicidal thoughts and behaviors among young adults (Healthy Minds Study, 2020–2021). All models are adjusted for gender, race/ethnicity, depression, anxiety, and food insecurity.

Figure 1 shows the synergy between loneliness and any interpersonal abuse (physical, emotional, or sexual) on the odds of suicidal ideation using an additive scale. Those who were only lonely had significantly greater odds of suicidal ideation (aOR: 3.01; 95% CI: 2.72–3.33), as did those who only had interpersonal abuse (aOR: 2.97; 95% CI: 2.52–3.50). However, those who endorsed both loneliness and interpersonal abuse had the greatest odds, exceeding the sum of the individual odds (the combined odds aOR: 5.65; 95% CI: 5.09–6.27). The ICR of 0.67 (95% CI: 0.14–1.20; *p* = 0.014) on an additive scale indicates that the combined odds of loneliness and interpersonal abuse is larger than the sum of the individual odds of the two exposures (i.e., 0.67 higher odds of suicidal ideation than if there were no synergy between loneliness and interpersonal abuse). Figure 1 shows a similar pattern using suicide plans as the outcome. Here, the synergy between loneliness and interpersonal abuse is evident, as the combined odds is greater than the sum of the individual odds (the combined aOR: 6.33; 95% CI: 5.31–7.56), with an ICR of 0.82 (95% CI: 0.13–1.53; *p* = 0.019). Figure 1 shows a similar pattern using suicide attempts as the outcome. Here, interpersonal abuse was associated with over four times greater odds of suicide attempts, whereas loneliness was associated with 1.78 times greater odds of suicide attempt. The synergy between loneliness and interpersonal abuse was also pronounced, as the combined odds was greater than the sum of the individual odds (the combined odds aOR: 8.49; 95% CI: 5.75–12.52), with a larger but non-significant ICR of 1.64 (95% CI: -0.18-3.45; *p* = 0.077).

## Discussion

In a sample of young adults in higher education in the US, loneliness and interpersonal abuse over the past year were both separately and synergistically associated with increased odds of suicidal ideation over the past year, even when accounting for sociodemographic factors, mental health (anxiety, depression), and socioeconomic status (food insecurity). A similar pattern emerged for suicide plans and suicide attempts, though only approaching statistical significance for suicide attempts when adjusting for sociodemographic characteristics and mental health. The association between interpersonal abuse and suicide attempts were stronger relative to suicidal ideation, potentially suggesting that the mechanism by which interpersonal abuse increases risk for STBs may operate through elevated capacity for suicide. To the best of the authors’ knowledge this is the first study to examine the synergy between loneliness and interpersonal abuse among young adults in higher education.

The synergy between loneliness and interpersonal abuse may represent a vicious cycle where socially isolated individuals (who have feelings of loneliness) are vulnerable to interpersonal abuse. For example, Eckhardt and colleagues [18] highlight literature that shows isolation can increase substance use, which in turn can increase the likelihood of conflicts and intimate partner violence. Conversely, interpersonal abuse can also result in symptoms of trauma, social isolation, and feelings of loneliness. Matthews and colleagues [34] for instance found that childhood victimization predicted loneliness in young adulthood. Further, studies have also shown that loneliness and isolation are common consequences of intimate partner violence [4]. The pathways that connect loneliness, interpersonal abuse, and STB are still emerging, and may involve a range of other factors not examined in this study.

Both loneliness and interpersonal abuse can have profound effect on brain structure and function, disrupting the hypothalamic-pituitary-adrenal axis, the serotonergic system, and the dopaminergic system, resulting in cognitive impairments (e.g., control of mood, pessimism, reactive aggressive traits, impaired problem solving, over-reactivity to negative social signs, negative pain), which may lead to STB [32, 48]). However, more research is needed to understand the neurobiological, cognitive, and psychosocial impacts of loneliness interacting with interpersonal abuse. As stated earlier, the ITS allowed us to cast loneliness as a marker of thwarted belongingness and perceived burdensomeness, while framing interpersonal abuse as a means of acquiring the capability for suicide. The vulnerability stress model further provided the rationale for testing for moderation, given that the link between psychological factors may depend on the presence of negative life events, which in this case meant that the association between loneliness and STB may be amplified by interpersonal abuse.

### Limitations and future directions

Findings should be interpreted considering several limitations. First, in terms of design, the data were cross-sectional and did not allow us to determine the temporal order of events to make any causal inferences. The relationships among loneliness, interpersonal abuse, and STB are difficult to disentangle. The relationships are likely bidirectional, as individuals with a history of STBs may experience heightened social withdrawal and loneliness (Heinrich & Gullone, 2006). Second, in terms of the sample, the study only examined students in higher education in the United States, limiting generalizability beyond this population. Future research can replicate our findings in more representative datasets. The HMS employed a convenience sampling strategy, which produced a large sample but with a relatively low response rate (14%). The response rate is typical for online convenience samples [3, 12], and we applied non-response survey weights to compensate consistent with prior studies using the dataset [33]; however, sampling bias remains a concern. Third, in terms of measurement, the interpersonal abuse items may not have captured the broad range of abuse that people can experience, nor their frequencies and severities. Thus, we may have underestimated the associations between interpersonal abuse and STB. Additionally, there may have been some social desirability bias in that students may have been reluctant to disclose loneliness, interpersonal abuse, or STB. At the same time, it is possible that people with mental, psychological, and emotional concerns may have selected into the study, resulting in higher prevalence of loneliness, interpersonal abuse, and STB. Finally, the data were collected in 2020–2021, during the first year of the pandemic, when loneliness and distress increased for students in higher education across the country [13, 16, 17, 42]. It is critical to corroborate the present findings using longitudinal data and data from other regions and populations outside of the context of the COVID-19 pandemic.

### Implications for prevention and intervention

While frameworks can elucidate suicide risk, it is still challenging to predict individual attempts, calling for more research into real-time risk and resilience assessment. The strong associations between loneliness, abuse, and STBs suggest a need for multifaceted prevention and intervention strategies. Several suicide prevention treatments have been developed [14, 25, 47], and among them, social support interventions, such as peer support groups and community-based programs, may help alleviate loneliness and provide emotional validation for survivors of abuse [8]. Trauma-informed practices [36], cognitive-behavioral therapy [47], and mindfulness-based interventions may also help prevent suicide, including among individuals with a history of abuse [29]. Additionally, campaigns aimed at reducing stigma surrounding mental health treatment on campuses are crucial to reduce feelings of thwarted belongingness and perceived burdensomeness, social isolation, and loneliness [51]. Institutions of higher education may consider implementing screening tools for loneliness and recent abuse to identify individuals at risk for STB, and connect people who screen positive with appropriate resources [7].

## Conclusion

The present study was among the first to test the individual and combined associations between loneliness and interpersonal abuse in relation to STB among young adults in higher education. Loneliness and abuse are interrelated risk factors for STBs, and while each factor independently contributes to suicide risk, their interaction may amplify vulnerability. People who experience interpersonal abuse while lonely or experience loneliness and social isolation following interpersonal abuse may be at particularly high risk, and future studies can explore whether social support interventions and abuse/trauma counseling together are especially efficacious in impacting suicide risk. Future research should further explore the nuanced mechanisms linking these factors and develop evidence-based strategies to enhance resilience and support systems.

## Supplementary Information

Below is the link to the electronic supplementary material.

Supplementary Material 1

## Data Availability

Availability of data and materials: Healthy Minds Study data are available upon request at: https://healthymindsnetwork.org/hms/.
