# Supplementary material for: The joint effects of loneliness and interpersonal abuse on suicidal thoughts and behaviors among young adults in higher education in the United States
Source: BMC Psychol. 2025 Jul 30;13:849. doi: 10.1186/s40359-025-03184-z (PMC12312561; doi:10.1186/s40359-025-03184-z)
Supplement: Supplementary file 1 — Supplementary Material 1 [file 40359_2025_3184_MOESM1_ESM.docx]

. svy, subpop (if age<30): logistic sui_idea anyabuse lonefinal lxa c.age i.gender i.raceethni

> city i.fin_foodinsec_flag i.dx_chronic_any

(running logistic on estimation sample)

Survey: Logistic regression

Number of strata = 1 Number of obs = 118,370

Number of PSUs = 140 Population size = 118,268.39

Subpop. no. obs = 101,972

Subpop. size = 99,480.132

Design df = 139

F(13, 127) = 243.06

Prob > F = 0.0000

-----------------------------------------------------------------------------------------

| Linearized

sui_idea | Odds ratio std. err. t P>|t| [95% conf. interval]

------------------------+----------------------------------------------------------------

anyabuse | 3.471693 .2563173 16.86 0.000 3.000161 4.017335

lonefinal | 4.514131 .2297831 29.61 0.000 4.081924 4.992103

lxa | .6614301 .0548482 -4.98 0.000 .561409 .7792711

age | .9647408 .0064492 -5.37 0.000 .9520735 .9775766

|

gender |

Woman | 1.010412 .0380098 0.28 0.783 .9379863 1.088429

Queer/non-binary | 2.521678 .1540328 15.14 0.000 2.2348 2.845382

|

raceethnicity |

Asian Pacific Islander | 1.017968 .0522385 0.35 0.729 .9197501 1.126674

Black | 1.013507 .0613695 0.22 0.825 .8991502 1.142407

Hispanic | .8307574 .0576742 -2.67 0.008 .7242053 .9529864

Two or More | 1.183056 .0613503 3.24 0.001 1.067767 1.310793

Other | .7507138 .0994687 -2.16 0.032 .5776977 .9755469

|

fin_foodinsec_flag |

Food insecure | 1.709767 .0518175 17.70 0.000 1.610324 1.815351

1.dx_chronic_any | 1.156691 .0354436 4.75 0.000 1.088693 1.228936

_cons | .0606484 .0093579 -18.16 0.000 .0447021 .0822832

-----------------------------------------------------------------------------------------

Note: _cons estimates baseline odds.

. nlcom exp(_b[anyabuse]+_b[lonefinal]+_b[lxa])-exp(_b[anyabuse])-exp(_b[lonefinal])+1

_nl_1: exp(_b[anyabuse]+_b[lonefinal]+_b[lxa])-exp(_b[anyabuse])-exp(_b[lonefinal])+1

------------------------------------------------------------------------------

sui_idea | Coefficient Std. err. z P>|z| [95% conf. interval]

-------------+----------------------------------------------------------------

_nl_1 | 3.379896 .3796855 8.90 0.000 2.635726 4.124066

------------------------------------------------------------------------------

. svy, subpop (if age<30): logistic sui_plan anyabuse lonefinal lxa c.age i.gender i.raceethni

> city i.anx i.dep i.fin_foodinsec_flag i.dx_chronic_any

(running logistic on estimation sample)

Survey: Logistic regression

Number of strata = 1 Number of obs = 115,598

Number of PSUs = 140 Population size = 115,323.64

Subpop. no. obs = 99,200

Subpop. size = 96,535.384

Design df = 139

F(15, 125) = 144.43

Prob > F = 0.0000

-----------------------------------------------------------------------------------------

| Linearized

sui_plan | Odds ratio std. err. t P>|t| [95% conf. interval]

------------------------+----------------------------------------------------------------

anyabuse | 3.017527 .316629 10.53 0.000 2.452168 3.713233

lonefinal | 2.631773 .2318213 10.99 0.000 2.211115 3.13246

lxa | .71305 .0836629 -2.88 0.005 .5654191 .8992272

age | .9513245 .0098958 -4.80 0.000 .9319587 .9710927

|

gender |

Woman | .8596984 .0483512 -2.69 0.008 .7692231 .9608153

Queer/non-binary | 1.751301 .1502072 6.53 0.000 1.478131 2.074955

|

raceethnicity |

Asian Pacific Islander | 1.25628 .0795354 3.60 0.000 1.108468 1.423801

Black | 1.238839 .1045395 2.54 0.012 1.048468 1.463775

Hispanic | .922218 .0721172 -1.04 0.302 .7901058 1.07642

Two or More | 1.240254 .0814281 3.28 0.001 1.089268 1.412169

Other | .7276429 .1448322 -1.60 0.112 .490913 1.078529

|

1.anx | 1.592966 .0919823 8.06 0.000 1.421098 1.78562

1.dep | 4.038749 .2896394 19.47 0.000 3.504828 4.654007

|

fin_foodinsec_flag |

Food insecure | 1.353573 .0573548 7.14 0.000 1.244793 1.471859

1.dx_chronic_any | 1.178687 .0545881 3.55 0.001 1.07555 1.291713

_cons | .0153307 .0037461 -17.10 0.000 .0094567 .0248533

-----------------------------------------------------------------------------------------

Note: _cons estimates baseline odds.

. nlcom exp(_b[anyabuse]+_b[lonefinal]+_b[lxa])-exp(_b[anyabuse])-exp(_b[lonefinal])+1

_nl_1: exp(_b[anyabuse]+_b[lonefinal]+_b[lxa])-exp(_b[anyabuse])-exp(_b[lonefinal])+1

------------------------------------------------------------------------------

sui_plan | Coefficient Std. err. z P>|z| [95% conf. interval]

-------------+----------------------------------------------------------------

_nl_1 | 1.013348 .3120007 3.25 0.001 .4018382 1.624858

------------------------------------------------------------------------------

. svy, subpop (if age<30): logistic sui_att anyabuse lonefinal lxa c.age i.gender i.raceethnic

> ity i.anx i.dep i.fin_foodinsec_flag i.dx_chronic_any

(running logistic on estimation sample)

Survey: Logistic regression

Number of strata = 1 Number of obs = 115,598

Number of PSUs = 140 Population size = 115,323.64

Subpop. no. obs = 99,200

Subpop. size = 96,535.384

Design df = 139

F(15, 125) = 54.81

Prob > F = 0.0000

-----------------------------------------------------------------------------------------

| Linearized

sui_att | Odds ratio std. err. t P>|t| [95% conf. interval]

------------------------+----------------------------------------------------------------

anyabuse | 4.406654 1.04911 6.23 0.000 2.752201 7.055662

lonefinal | 2.520064 .4884764 4.77 0.000 1.717789 3.697032

lxa | .6913084 .1714841 -1.49 0.139 .4233218 1.128946

age | .901886 .0182847 -5.09 0.000 .8664489 .9387723

|

gender |

Woman | .928932 .0926981 -0.74 0.461 .7625995 1.131544

Queer/non-binary | 1.640243 .2531135 3.21 0.002 1.208931 2.225434

|

raceethnicity |

Asian Pacific Islander | 1.612599 .2401173 3.21 0.002 1.201347 2.164632

Black | 1.82732 .3444659 3.20 0.002 1.25877 2.652667

Hispanic | 1.144306 .1720546 0.90 0.372 .8500292 1.54046

Two or More | 1.337694 .1621524 2.40 0.018 1.052616 1.69998

Other | 1.155532 .4155785 0.40 0.688 .5674989 2.352875

|

1.anx | 1.479236 .1451643 3.99 0.000 1.218348 1.795988

1.dep | 3.190646 .4760405 7.78 0.000 2.375554 4.285409

|

fin_foodinsec_flag |

Food insecure | 1.800332 .1713903 6.18 0.000 1.491445 2.173192

1.dx_chronic_any | 1.323805 .1364768 2.72 0.007 1.079691 1.623114

_cons | .0074614 .003534 -10.34 0.000 .002925 .0190335

-----------------------------------------------------------------------------------------

Note: _cons estimates baseline odds.

. nlcom exp(_b[anyabuse]+_b[lonefinal]+_b[lxa])-exp(_b[anyabuse])-exp(_b[lonefinal])+1

_nl_1: exp(_b[anyabuse]+_b[lonefinal]+_b[lxa])-exp(_b[anyabuse])-exp(_b[lonefinal])+1

------------------------------------------------------------------------------

sui_att | Coefficient Std. err. z P>|z| [95% conf. interval]

-------------+----------------------------------------------------------------

_nl_1 | 1.750296 .850836 2.06 0.040 .082688 3.417904

------------------------------------------------------------------------------

.

.
